# NMR Detection and Structural Modeling of the Ethylene Receptor LeETR2 from Tomato

**DOI:** 10.3390/membranes12020107

**Published:** 2022-01-18

**Authors:** Shukun Wei, Yaqing Yang, Yuan Yuan, Lingyu Du, Hongjuan Xue, Bo OuYang

**Affiliations:** 1 State Key Laboratory of Molecular Biology, Shanghai Institute of Biochemistry and Cell Biology, CAS Center for Excellence in Molecular Cell Science, Chinese Academy of Sciences, Shanghai 200031, China; weishukun2017@sibcb.ac.cn (S.W.); yangyaqing2015@sibcb.ac.cn (Y.Y.); yuanyuan03@sibs.ac.cn (Y.Y.); dulingyu@sibcb.ac.cn (L.D.); 2 University of Chinese Academy of Sciences, Beijing 100049, China; 3 National Facility for Protein Science in Shanghai, Zhangjiang Laboratory, Shanghai Advanced Research Institute, Chinese Academy of Sciences, Shanghai 201203, China; xuehongjuan@sari.ac.cn

**Keywords:** LeETR2, AlphaFold2, membrane protein expression, solution NMR, organic solvent, molecular docking, molecular dynamics simulation

## Abstract

The gaseous plant hormone ethylene influences many physiological processes in plant growth and development. Plant ethylene responses are mediated by a family of ethylene receptors, in which the N-terminal transmembrane domains are responsible for ethylene binding and membrane localization. Until now, little structural information was available on the molecular mechanism of ethylene responses by the transmembrane binding domain of ethylene receptors. Here, we screened different constructs, fusion tags, detergents, and purification methods of the transmembrane sensor domain of ethylene receptors. However, due to their highly hydrophobic transmembrane domain (TMD), only a KSI-fused LeETR2_1–131_ from tomato yielded a good-quality nuclear magnetic resonance (NMR) spectrum in the organic solvent. Interestingly, a dimer model of LeETR2_1–131_ built by the AlphaFold2 algorithm showed greatly converged structures. The interaction analysis of ethylene and LeETR2_1–131_ using molecular docking and molecular dynamics (MD) simulations demonstrated the potential binding sites of ethylene in LeETR2_1–131_. Our exploration provides valuable knowledge for further understanding of the ethylene-perception process in ethylene receptors.

## 1. Introduction

Gaseous ethylene is a plant hormone that controls numerous processes in plant growth and development, including seedling growth, leaf and flower senescence, fruit ripening, and plant responses to pathogens [1,2,3]. Many reactions to ethylene are initiated by a group of endoplasmic reticulum (ER)-membrane bound receptors. These ethylene receptors are negative regulators of ethylene responses [4], which switch off the downstream signal transmission upon ethylene binding, and hence mediate the plant growth and development [5]. Earlier studies have demonstrated that the ethylene receptors form a complex with constitutive triple response 1 (CTR1) and ethylene insensitive 2 (EIN2) in the response to the plant hormone [6]. In the absence of ethylene, the ethylene receptor is active, and triggers CTR1 on for inhibiting the plant development; when ethylene binds, the ethylene receptor is inhibited and turns CTR1 off, resulting in the initiation of downstream signaling [7]. A number of inhibitors, including Ag^+^ and some gas molecules, have been reported to block ethylene receptor action to improve the shelf life of fruits, vegetables, and flowers [8]. The essential roles of ethylene receptors in plant growth and development uncover a great potential of ethylene receptors as a target for practical use in agriculture and horticulture in the future.

Sequence analysis and functional studies of ethylene receptors suggest that ethylene receptors are similar to bacterial two-component histidine kinase receptors [9]. In *Arabidopsis thaliana*, the five ethylene receptors, comprising ETR1, ERS1, ETR2, ERS2, and EIN4, can be classified into two subfamilies, in which ETR1 and ERS1 form the type-I subfamily, and the type-II subfamily contains ETR2, ERS2, and EIN4 [10]. All members of the receptor family are composed of an N-terminal transmembrane domain (TMD), a middle GAF domain, and a C-terminal catalytic histidine kinase (HK) domain, displaying a similar overall modular structure. ETR1 and ERS1 in subfamily I contain three transmembrane helices, while the subfamily II members have an additional fourth transmembrane helix. The basic functional unit of the ethylene receptors is a disulfide-linked dimer [11]. The N-terminal TMD is the sensor domain that binds copper ions, and hence binds the ethylene molecules selectively and noncovalently, which is different from the majority of membrane receptors that have a soluble signal-binding domain [12].

Until today, the ethylene binding of the receptors and the inhibition mechanism in the ethylene signaling network were still obscure. Previously, the structures of the ethylene receptors ERS1 and ETR1 from *Arabidopsis thaliana (At)*, including GAF, catalytic ATP binding, DHp, and receiver domains, have been obtained by X-ray crystallography and low-resolution, small-angle X-ray scattering (SAXS) [12]. Together, this has allowed obtaining a model of the entire cytosolic domain. The high-resolution structure of the transmembrane sensor domain (TSD) is yet unknown; a structural model of AtETR1_TSD was recently generated by combining *ab initio* protein structure prediction and a coevolutionary relationship [13]. A dimeric model of AtETR1_TSD with copper(I) was further built based on the experimentally determined copper stoichiometry. These models provided insights into the helix assembly and dimerization for ETR1_TSD [13]. However, how ethylene receptors are inhibited by sensing ethylene and the conformational change of the dimeric ethylene receptors in response to the inhibitors are still unknown. Therefore, further high-resolution structural studies are needed to understand the mechanistic details of the activation and inhibition of ethylene action.

To determine the structure of TSD for ethylene receptors, here we screened the expression and purification of the ethylene receptor’s TSDs in *Escherichia coli* (*E. coli)*. Different constructs, fusion tags, and detergents were examined, and only the TSD (residues 1–131) from tomato ETR2 (LeETR2) was successfully expressed in the *E. coli* strain BL21 (DE3) as inclusion bodies, and achieved high yield and purity by Ni-NTA affinity chromatography and high-performance liquid chromatography (HPLC). Unfortunately, only in the organic solvent hexafluoroisopropanol (HFIP) did we obtain a good-quality NMR spectrum for LeETR2_1__–131_. Benefiting from the great power of AlphaFold2, we built a dimer model of LeETR2_1__–131_, which was highly converged and rigid. Molecular dynamics (MD) simulations of LeETR2_1__–131_ with ethylene offered the potential binding sites of ethylene, which shed light on the relationship between the TSD structure and the ethylene binding.

## 2. Materials and Methods

### 2.1. Materials

#### 2.1.1. Reagents

Ammonium chloride (^15^N, 99% (*w*/*w*); Cat. NLM-467), D_2_O (99.96% (*v*/*v*; Cat. DLM-6-PK), and HFIP (99.5%, Cat. H107501) were obtained from Aladdin (Shanghai, China). Other chemicals were sourced from Amresco (Solon, OH, USA), Sigma-Aldrich (St. Louis, MO, USA), and Sangon Biotech (Shanghai, China).

#### 2.1.2. Medium for Cell Culture

Luria–Bertani (LB) (1 L): 10 g tryptone, 5 g yeast extract, 10 g NaCl with 100 μg/mL ampicillin. ^15^N M9 medium (1 L): 6 g Na_2_HPO_4_, 3 g KH_2_PO_4_, 1 g NH_4_Cl (N, 99%, for ^15^N-labeled samples), 0.5 g NaCl, 4 g glucose, 1 mL 2 M MgSO_4_, 1 mL 100 mM CaCl_2_, 4 mL Centrum stock solution (1 tablet of Centrum dissolved in 40 mL ddH_2_O then filtered).

### 2.2. Construct Design

To obtain better dissolved target ethylene receptor peptides, we calculated the individual grand average of hydropathicity (GRAVY) (ProtParam tool. Available online: web.expasy.org/protparam, accessed on 18 December 2021) for every curated peptide sequence from the ethylene receptor family. Four peptide sequences (AtETR1, AtETR2, AtERS2, and LeETR2) were chosen according to their lower GRAVY values, which meant better solubility for the sample preparation. Meanwhile, His8-tag, His9-trpLE, MBP tag, and His8-KSI (ketone steroid isomerase) tag were used for the overexpression, individually.

Synthesized oligonucleotides (Genscript. Available online: www.genscript.com.cn, accessed on 29 December 2021) corresponding to the membrane-spanning sequence of AtETR1 (*Arabidopsis*) (Uniprot ID: P49333), AtETR2 (*Arabidopsis*) (Uniprot ID: Q0WPQ2), AtERS2 (*Arabidopsis*) (Uniprot ID: P93825), and LeETR2 (*Tomato*) (Uniprot ID: O49187) were optimized to *E. coli* codons according to the preferred protein translation codon usage in *E. coli*.

### 2.3. Protein Expression

The above constructed plasmids were transformed into BL21 (DE3) cells and plated on LB medium supplemented with appropriate ampicillin resistance, then grown in an incubator at 37 °C overnight.

A single colony of the transformant was inoculated into 5 mL of LB and 100 μg/μL ampicillin. The culture was put in a shaker at 37 °C and shaken at 220 rpm for 8 h. Then, 50 μL of the grown cells were added to 200 mL LB media to continue the growth at 37 °C with shaking at 220 rpm overnight. Then, the cells were spun down and inoculated into 1 L of sterile ^15^N labelled M9 media in a 3 L baffled flask. The expression of the target proteins was induced by adding 0.2 mM isopropyl-β-D-thiogalactopyranoside (IPTG) at OD600 = 0.7. Then, the cells were grown at 20 °C for 18 h. Finally, the cells were harvested by centrifugation at 4000 rpm for 30 min at room temperature (Avanti J-20XP Centrifuge, Beckman JLA-8.1000 rotor, Beckman Coulter, Brea, CA, USA).

### 2.4. Protein Purification

The successfully overexpressed LeETR2_1–131_ with a His8-KSI fusion tag was extracted from the inclusion bodies and solubilized in 6 M guanidine HCl, 200 mM NaCl, 1% Triton X-100, and 50 mM Tris-HCl pH 8.0 (Buffer A). The His8-KSI-LeETR2_1–131_ fusion protein was loaded to Ni^2+^ affinity (NTA) column in Buffer A at room temperature and eluted from the NTA column in the same buffer with 500 mM imidazole. The eluted fusion protein was then cleaved by cyanogen bromide (CNBr) in 80% formic acid (FA) for 1 h to remove the KSI tag. The cleaved mixtures were further loaded onto a Proto-C18 column (Agilent Technology, Santa Clara, CA, USA). A reverse-phase chromatography using the gradient of 55–96% buffer B (100% acetonitrile (ACN) + 0.1% trifluoroacetic acid (TFA)) was performed to obtain highly pure LeETR2_1–131_. NMR samples were prepared by dissolving 1.5 mg of lyophilized peptide in organic solvent (60%HFIP + 30%ddH_2_O + 10%D_2_O).

### 2.5. SDS-PAGE

All the proteins were examined using the Tris–Tricine SDS-PAGE system following a previous protocol [14]. The samples were mixed with the gel loading buffer and β-mercaptoethanol (β-ME), and stained with Coomassie Blue G250 after the electrophoresis.

### 2.6. Mass Spectrometry

Approximately 0.2 mg of lyophilized LeETR2_1–131_ was dissolved in 10 μL 50% formic acid (FA), and 1 μL of this protein solution was mixed with 1 μL of matrix solution (10 mg/mL sinnapinic acid (SA), 75% ACN, 25% H_2_O, 0.1% TFA). The resulting solution was spotted onto a seed layer spot on the MALDI target. The mass spectrum was collected on a 5800 MALDI-TOF/TOF (Applied Biosystems, Waltham, MA, USA).

### 2.7. NMR Detection for LeETR2_1–131_

Samples were prepared by dissolving 1–2 mg of ^15^N-labelled protein in 500 μL of NMR buffer (60%HFIP + 30%ddH_2_O + 10%D_2_O). The solution NMR experiments were performed on an Agilent 700 MHz spectrometer equipped with a triple-resonance 5 mm probe. The two-dimensional (2D) ^1^H/^15^N TROSY-HSQC spectra were obtained at 30 °C. The NMR data were processed using NMRPipe and rendered in NMRDraw on a Dell Precision T7810 Linux workstation [15].

### 2.8. AlphaFold2 modeling and relaxation

No structure of LeETR2 was available in the PDB database (PDB. Available online: https://www.pdbus.org/, accessed on 2 December 2021), so the structural model for LeETR2_1–131_ was thus calculated using AlphaFold2 [16] (AlphaFold2. Available online: https://github.com/deepmind/alphafold, accessed on 11 October 2021). No homologous templates were used; only the multiple sequence alignment (MSA) was set as true, and the MSA mode was MMseq2. The model procedure was under a homo-dimer construction. The residue confidence score provided by AlphaFold2 (named pIDDT) was above 80% for most residues of LeETR2_1–131_. For residues in the alpha-helix regions, pIDDT values were above 90% nearly for all. After analysis, it was shown that the copper binding site containing C64 and H68 of LeETR2_1–131_ in the model was well inward-facing inside the channel, and the alpha-helix was well packed. The relaxation of the unrelaxed model of LeETR2_1–131_ was conducted using a built-in algorithm for removing the steric clashes of side-chains and incorporating all the hydrogens. Through Ramachandran analysis of these two relaxed models, it was shown that almost all the residue atoms were located in the most favored or allowed regions. Thus, the relaxed model of LeETR2_1–131_ was qualified for further docking and MD simulations.

### 2.9. Molecular Docking Using Autodock 1.2.2

Molecular docking of the ethylene was implemented in the LeETR2_1–131_ relaxed model. The two cofactor monovalent copper ions were placed around the copper binding sites, C64 and H68 of LeETR2_1–131_. The receptor of LeETR2_1–131_ was protonated using the REDUCE algorithm and prepared using the prepare-receptor algorithm, all from the ADFR suite [17]. The ethylene was protonated and prepared using the mk-prepare-ligand algorithm from the ADFR suite [17] (ADFR suite. Available online: https://ccsb.scripps.edu/, accessed on 2 December 2021). The Autodock Vina 1.2.2 software [18] (Autodock Vina 1.2.2. Available online: https://github.com/ccsb-scripps/AutoDock-Vina, accessed on 2 December 2021) was utilized for molecular docking, in which vinardo scoring function parameters were selected. The grid dimensions were set to 30 × 30 × 30 Å to allow enough regions to cover the copper binding sites, where the coordinate of the grid center was set to 0 × 0 × 5.844 Å (LeETR2_1–131_). In addition, the default values were used for other parameters with the exhaustiveness of 32 for better searching space. The best-fitted poses from docking models were used for further MD simulations.

### 2.10. Molecular Dynamics Simulation Using Desmond

The MD simulations of LeETR2_1–131_ were performed using the Desmond package [19] (Desmond. Available online: https://www.deshawresearch.com/, accessed on 2 December 2021) in the presence or absence of ethylene with copper ions. LeETR2_1–131_ was placed in a 1-palmitoyl-2-oleoyl-sn-glycero-3-phosphocholine (POPC) lipid bilayer with an appropriate amount of counterions to balance the net charge of the system solvated in 0.15 M KCl. The membrane of LeETR2_1–131_ dimeric complex was defined through helices. The whole system was solvated with the explicit simple point charge (TIP3P) waters under the force field OPLS2005 [20]. The simulation temperature (300 K) was controlled by Nose–Hoover temperature coupling [21], and the atmospheric pressure (1 atm) was modulated by the Martina–Tobias–Klein method [22]. The particle-mesh Ewald (PME) method [23] was used to calculate electrostatic interactions and van der Waals (VDW) forces. The system was relaxed before the simulation runs using the default setting in Desmond. The initial coordinates of LeETR2_1–131_-C_2_H_4_ for the MD simulation calculations were taken from docking results. The system was subject to 100 ns of a normal pressure and temperature (NPT) production simulation run.

## 3. Results

### 3.1. Protein Expression of Ethylene Receptors

**Figure 1:**
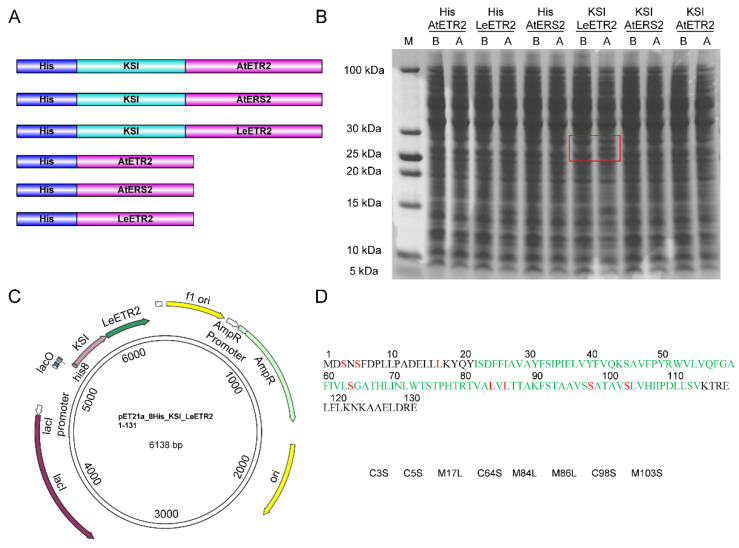
The construct design and expression of ethylene receptors. (**A**) The His8-tagged KSI constructs and His8-tagged constructs of ETR2 or ERS2. For each protein construct, different colors represent different domains: His8 tag (blue), KSI (cyan), protein sequence (pink). (**B**) The screening expression results of 6 constructs. (**C**) One example of the plasmid map of His8-KSI tagged LeETR2_1–131_ construct used for expression. (**D**) The amino acid sequences of LeETR2_1–131_ and the mutation sites with respect to the purification.

In the initial trials, we tested the expression and purification of ETR1_TMD from *Arabidopsis thaliana* (AtETR1, ID: P49333). We synthesized and cloned five different constructs with trpLE, MBP, and GFP tags fused to the target sequence AtETR1_1–116_. Only using MBP as a fusion partner, AtETR1_1–116_ was successfully overexpressed in inclusion bodies. However, the MBP-cleaved product could not be well dissolved for further purification, indicating the high hydrophobicity of AtETR1_1–116_. We also noticed that MBP structures were destroyed in the high content of detergents, which presented difficulties in separating AtETR1_1–116_ from unfolded MBP. To find a target sequence with better solubility, we calculated the GRAVY values (indicator of hydrophobicity) of 26 ethylene receptors using ProtParam (ProtParam tool. Available online: https://web.expasy.org/protparam/, accessed on 18 December 2021). The top three constructs (AtETR2, AtERS2, LeETR2) with the lowest GRAVY values were used for plasmid construction. In addition, these plasmids were inserted into the expression vectors with His8-KSI tag or only His8-tag for expression screening (Figure 1A). Cysteines were removed in all sequences to simplify the purification procedures. For KSI-fused constructs, methionines were further mutated, since methionine was the cleavage site for cyanogen bromide (CNBr) in the following purification step. The expression test showed that only LeETR2_1–131_ with His8-KSI tag was expressed successfully with 0.2 mM IPTG at 20 °C, with an expression band appearing (~29 kDa) in Coomassie blue staining gel (Figure 1B), which was used for further expression and purification (Table S1).

The recombinant LeETR2_1–131_ with a KSI fusion tag (Figure 1C) was expressed in inclusion bodies and thus protected from proteolysis, but required reconstitution for solution NMR studies. Therefore, cysteines in LeETR2_1–131_ were mutated into serines at positions 3, 5, 64, and 98 to prevent the formation of disulfide bonds during reconstitution. Meanwhile, methionines at positions 17, 84, 86, and 103 were mutated to leucine or serine to avoid the side reactions of CNBr cleavage in the following purification steps (Figure 1D). These mutations were implemented according to the sequence conservation and similarity to maintain the activity of LeETR2_1–131_ (Figure S1).

### 3.2. LeETR2_1–131_ Purification and NMR Detection

**Figure 2:**
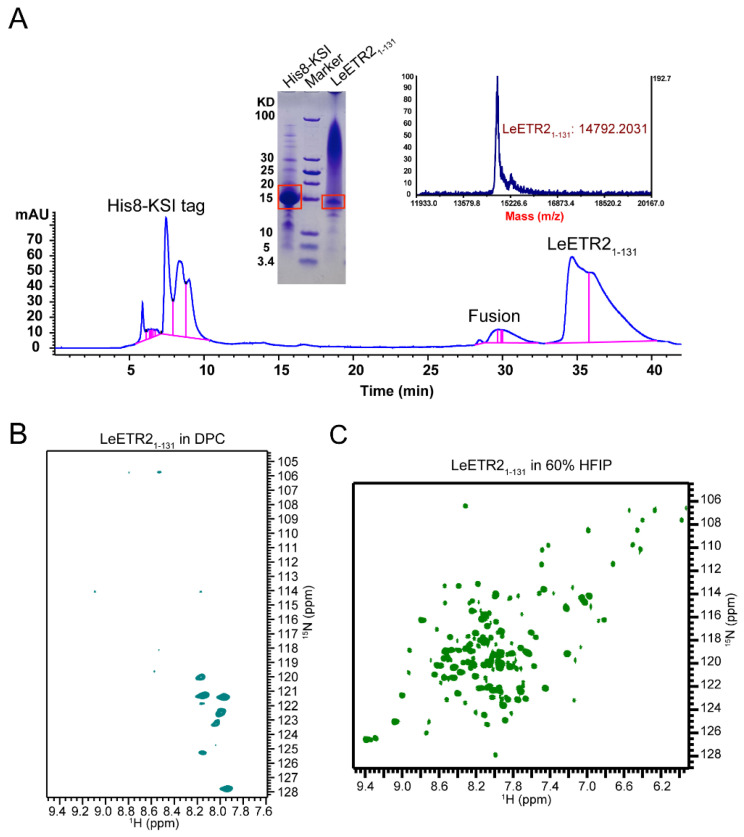
The expression and NMR detection of LeETR2_1–131_ using the Bruker AVANCE 600 MHz spectrometer. (**A**) The HPLC plot of purification procedure for LeETR2_1–131_, which were detected using mass spectrometry and SDS-PAGE. (**B**) The NMR TROSY-HSQC spectrum of LeETR2_1–131_ in DPC micelles, which was purified from reversed-phase HPLC. The sample could not be well dissolved in this type of detergent. (**C**) The NMR TROSY-HSQC spectrum of LeETR2_1–131_ in 60% HFIP. The sample could be well dissolved in this type of organic solvent compared with given detergents.

The detailed purification of LeETR2_1–131_ proteins was described in the above methods. Briefly, the LeETR2_1–131-_KSI fusion protein was overexpressed in inclusion bodies and resuspended in 6 M guanidine buffer with 1% Triton X-100 before being loaded to the nickel affinity column. The bound protein was washed then eluted by the elution buffer with 500 mM imidazole. The LeETR2_1–131_ protein was then cleaved by a methionine site-specific cleavage using CNBr. The final purification step was performed by reverse-phase (RP)-HPLC in a C18 column. A typical elution profile is shown in Figure 2A. Three major peaks corresponding to the expected cleavage products of His8-KSI, fusion, and LeETR2_1–131_ were independently pooled and detected by MALDI-TOF mass spectrometry, which gave a mass 14792.2 Da for the fraction of LeETR2_1–131_ (Figure 2A), very close to the theoretical molecular weight of 14819.08 Da. The identification of the purified proteins was confirmed by SDS-PAGE (Figure 2A). This purified recombinant protein was further used for NMR studies. Typically, 3 mg of purified protein could be obtained from 1 L of cell culture in ^15^N-labelled M9 minimal media.

Pure LeETR2_1–131_ was first solubilized in the detergents dodecylphosphocholine (DPC), dihexanoylphosphatidylcholine (DHPC), lyso-myristoyl phosphatidylglycerol (LMPG), and sodium dodecylsulphate (SDS); however, these initial trials failed to provide high-quality NMR spectra. Very few signals were recorded in the DPC micelle system (Figure 2B). Meanwhile, the proteins reconstituted in the classic 1,2-Dimyristoyl-sn-glycero-3-phosphorylglycerol (DMPG)/DHPC or 1,2-Dimyristoyl-sn-glycero-3-phosphocholine (DMPC)/DHPC lipid bicelles at q = 0.3, 0.5, 0.7 also behaved poorly. The solubility screening of LeETR2_1–131_ showed that a good solubility of LeETR2_1–131_ was reached in 60% HFIP, which gave a dispersed spectrum with an appropriate number of resonances at 303 K on a spectrometer with a ^1^H resonance frequency of 600 MHz (Figure 2C). Each resonance represented a single ^15^N-labelled site of the protein, and the limited chemical shift dispersion indicated that LeETR2_1–131_ maintained some helical structures in 60% HFIP. However, considering the strong effects of organic solvents on disrupting the protein tertiary structure and receptor activity, further structural studies of LeETR2_1–131_ were not pursued in this organic solvent.

### 3.3. LeETR2_1–131_ Protein Modeling

**Figure 3:**
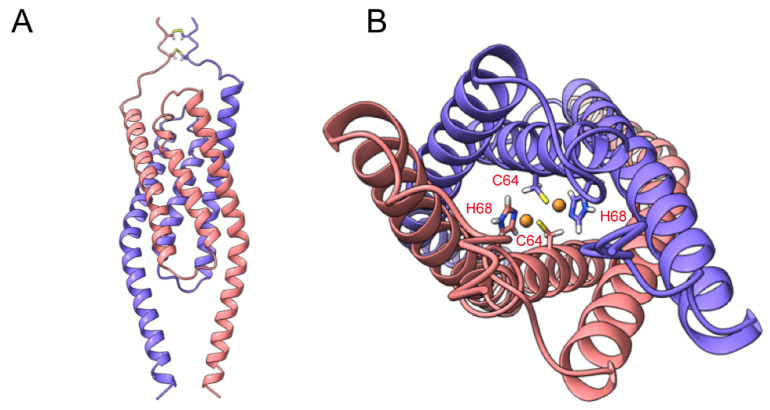
The protein modeling of LeETR2_1–131_ using AlphaFold2. (**A**) The side view of the dimeric model of LeETR2_1–131_ computed from AlphaFold2. (**B**) The top view of LeETR2_1–131_ displaying the orientation of copper ions and copper binding sites C64 and H68, which are facing inward. The individual copper binding sites C64 and H68 of LeETR2_1–131_ are depicted as sticks.

To further understand the structure and ethylene binding of LeETR2_1–131_, we performed the *ab initio* modeling of LeETR2_1–131_ (Uniprot: O49187) using AlphaFold2 [16] (AlphaFold2. Available online: https://github.com/deepmind/alphafold, accessed on 11 October 2021) with MMseqs2 (UniRef+Environmental) for the mode of multiple sequence alignment (MSA). The restriction parameters were set as the defaults, and the oligomer state was set as a dimer. The evaluation of models of LeETR2_1–131_ were qualified using a Ramachandran plot (Figure S2). The model quality was improved after relaxation, with almost all residues located in the most favored region. The distribution of the five generated LeETR2_1–131_ models (Figure S3) showed that these dimeric models converged well with respect to the predicted alignment error (PAE) and the predicted local distance difference test (pIDDT). The representative structure of the obtained models revealed a three-helical arrangement, indicating that LeETR2_1–131_ resembled the AtETR1 structure that belongs to the type-I subfamily. The N-terminal part displayed a flexible region, with two disulfide bonds in-between chains formed by residues 3 and 5, respectively (Figure 3A). The C-terminal part formed a long helix connecting the transmembrane domain. The C-termini of the dimer were at opposite positions of the dimeric LeETR2_1–131_ assembly. Acquired models of LeETR2_1–131_ were unrelaxed; thus, the further built-in MD simulation was utilized for relaxing the side-chain clashes. LeETR2_1–131_ maintained the three TM helices and a rigid helical structure for the membrane-proximal region at the C-terminus after relaxation (Figure 3).

### 3.4. Molecular Docking of Ethylene into LeETR2_1–131_ around the Copper Binding Sites

**Figure 4:**
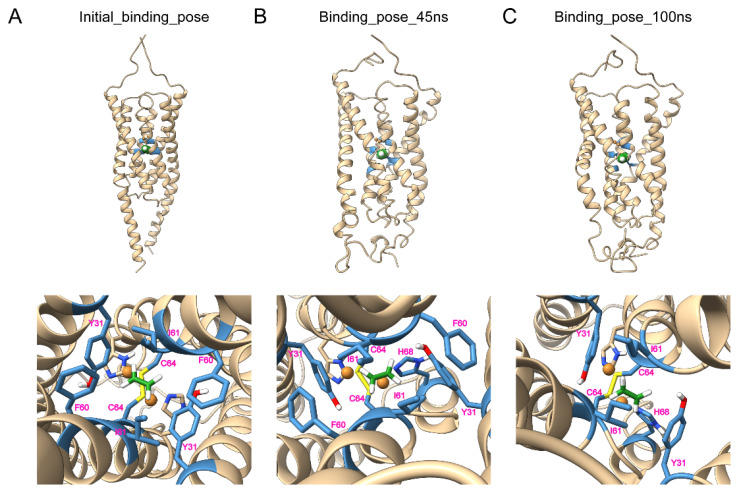
The molecular dynamics simulation of LeETR2_1–131_-C_2_H_4_. (**A**) The initial docking model of LeETR2_1–131_-C_2_H_4_ in the presence of Cu^+^ using molecular docking. The initial binding region was set around the copper binding sites C64 and H68 of LeETR2_1–131_. The calculated binding sites were Y31, F60, I61, and C64. (**B**) The molecular dynamics simulation of LeETR2_1–131_-C_2_H_4_ in the presence of Cu^+^ at a time of 45 ns; the MD trajectory was typically stable, and the binding sites were still stabilized at the sites of Y31, F60, I61, C64, and H68. (**C**) The molecular dynamics simulation of LeETR2_1–131_-C_2_H_4_ in the presence of Cu^+^ at a time of 100 ns. The snapshot shows that the binding conformation did not change very much; the interaction sites were located at Y31, I61, C64, and H68. The Cu^+^ ions are shown as spheres in bronze. The ethylene (green) or hydrogens (white) are shown as spheres or sticks. The chains of LeETR2_1–131_ are in gold, and the binding sites are colored in steel blue.

The LeETR2_1–131_/Cu dimer model was further generated with the 1:1 stoichiometry of LeETR2_1–131_:Cu in the light of previous studies [13]. To generate this model, ethylene was docked into the TMD model using Autodock Vina 1.2.2 [18] (Autodock Vina 1.2.2. Available online: https://github.com/ccsb-scripps/AutoDock-Vina, accessed on 2 December 2021). The molecular docking was used for creating an initial conformation of ethylene binding in the presence of two monovalent copper ions. The putative copper binding sites, composed of residues C64 and H68, as previously mentioned and suggested, were buried in the dimerization interface. The two monovalent copper ions were then placed around the copper binding sites at C64 and H68 of LeETR2_1–131_. The ethylene and LeETR2_1–131_ were protonated and converted into PDBQT format. The ethylene was docked around the copper binding sites C64 and H68 inside the receptor, and the initial binding sites of ethylene were Y31, F60, I61, and C64 (Figure 4A). This well-packed docking conformation was used for further MD simulation.

### 3.5. Molecular Dynamics Simulation of LeETR2_1–131_ with Cu^+^ and C_2_H_4_

The best-posed docking conformation of LeETR2_1–131_-ethylene was further used for MD simulation. The MD system consisted of a POPC bilayer membrane, TIP3P water models, and 150 mM KCl. To acquire the difference between the LeETR2_1–131_ status in the presence and absence of ethylene, MD without ethylene and MD with ethylene were performed, respectively. The simulation results showed that LeETR2_1–131_ in the presence or absence of ethylene had high stability, as the Cα and side-chain root-mean-square deviation (RMSD) in both simulations were lower than 5 angstroms. Moreover, LeETR2_1–131_ with ethylene had a lower mean RMSD than that of the apo state, indicating that the entire inhibited model was in a much more stable conformation (Figure S4A). The ligand-RMSD of ethylene for LeETR2_1–131_ showed that the position of ethylene did not change much, and remained stable (Figure S4B). The N- and C-termini of LeETR2_1–131_ showed the flexibility through simulation, while the root-mean-square fluctuation (RMSF) of Cα and side-chains of LeETR2_1–131_ in the apo state changed a bit more than that of the ethylene binding state (Figure S4C,D).

After 100 ns of MD simulation with ethylene, the representative binding poses were extracted every 5 ns. The comparison between each pose suggested that the binding sites were stabilized in the same region over the whole simulation. The snapshot at 45 ns with the most potential interaction sites and the final snapshot at 100 ns were drawn as representative binding modes. The interaction of ethylene with LeETR2_1–131_ at 45 ns was involved in the sites of Y31, F60, I61, C64, and H68, while C64 and H68 formed the interaction with copper ions (Figure 4B). In addition, the binding sites of ethylene with LeETR2_1–131_ at 100 ns were Y31, I61, C64, and H68, with interaction of C64 and H68 over copper ions (Figure 4C). These sites played a significant role in the ethylene binding and the ethylene perception at the beginning of signaling.

## 4. Discussion

In the long term, how the plant hormone ethylene is perceived by ethylene receptors and hence regulates the downstream signaling transduction lacks structural visualization. Here, we screened the ETR homologues in the *E. coli* expression system for determination of the atomic-level-resolution structure. However, due to the high hydrophobic contents, most of the screened constructs failed to produce a large amount of proteins. The available LeETR2_1–131_ proved to be very difficult to reconstitute in detergents for NMR study. Only in organic solvent was an NMR spectrum with good resolution and dispersion available. Therefore, we built a structural model by means of the AlphaFold2 algorithm. Alphafold2 is a recently published powerful computational method to predict protein structures from a protein sequence. To improve the accuracy of protein structure prediction, this method was coupled with existing structure and sequence databases deposited by the experimental community. The model from Alphafold2 displayed that LeETR2_1–131_ was an ETR1-like structure with three transmembrane helices, in which H1, H2, and H3 were located in the interface. A previous ETR1-TMD model of *Arabidopsis* *thaliana* (AtETR1-TMD) built by integrating *ab initio* Rosetta structure prediction and coevolutionary methods showed that H1 and H2 were the interface helices. The alignment of the LeETR2_1–131_ and AtETR1-TMD models showed large differences with RMSD 13.089 Å. The helices in the AtETR1-TMD model were straight and parallel to the center symmetric axis, while the helices in the LeETR2_1–131_ model were tilted with a 25° angle to the axis (Figure S5). These differences could have been caused by the sequence or homologue differences, or the prediction algorithm. Further experimental investigations are required to clarify these differences.

The ethylene binding model showed small differences between the initial model and after the MD simulation with the RMSD 3.106 Å (Figure 4). The largest changes came from the membrane-proximal region at the C-terminus, which lost its helical structure and became a flexible loop; as a result, it provided the flexibility of the membrane-proximal region to interact with the membranes. The top of the potential ethylene binding site in the model contained C64 and H68 to bind with copper ions, and the bottom of the binding site consisted of large hydrophobic residues Y31, F60, and I61; thus together, they could catch and lock the small gas molecule ethylene in the binding pocket tightly, which was consistent with previous results showing that AtETR1 had a very low *K*_d_ at 2.4 nM [24] or a dissociation constant of the response (*K*_r_) of 0.1 μL/L [25] to ethylene. In accordance, an earlier study mutated the corresponding binding residues of AtETR1, including Y32A, F61A, I62A, C65Y, and H69A, and led to the diminishment of ethylene binding activity [26]. The ethylene binding residues (Y31, I61, C64, and H68) of LeETR2_1–131_ maintained their binding to ethylene through the whole MD simulation, while F60 did not appear to interact with ethylene at the last snapshot, possibly due to the dynamics. The ethylene binding model of LeETR2_1–131_ after the MD simulation showed slightly larger differences from the apo model after the MD simulation with the RMSD 3.954 Å (Figure S6), which also displayed obvious changes at the C-terminal parts, including part of the transmembrane helices, indicating the C-terminal part was more dynamic during the signaling transduction.

Collectively, our exploration of the expression and purification of the ethylene receptors provided preliminary information for the structural studies of ethylene receptors. The dimeric model of LeETR2_1–131_ built by the AlphaFold2 algorithm [16] provided a good template for further molecular docking and MD simulations to decipher the potential binding sites of ethylene. The identified key binding residues of ethylene in LeETR2_1–131_ agreed well with previous findings in the ethylene receptor homologues. Our data also provided a comprehensive insight into subtle conformational changes of LeETR2_1–131_ in the presence of ethylene, which provided clues to understand the activity of ethylene receptors and enhance the activator/inhibitor development of ethylene receptors in the future, which is particularly important from both ecological and agricultural points of view.

## Data Availability

The data presented in this study are available in this article or supplementary materials.

## Supplementary Materials

The following are available online at https://www.mdpi.com/article/10.3390/membranes12020107/s1, Table S1: Expression conditions of different constructs, Figure S1: The alignment of ethylene receptor family, Figure S2: The evaluation of unrelaxed and relaxed models of LeETR2_1–131_ using AlphaFold2, Figure S3. The evaluation of models of LeETR2_1–131_ from AlphaFold2, Figure S4. The Cα and side-chain RMSD of LeETR2_1–131_ in the absence or presence of ethylene, Figure S5. The comparison of LeETR2_1–131_ and AtETR1_1–117_ models, Figure S6: The comparison of LeETR2_1–131_ in the absence or presence of ethylene binding.

## Sample Availability

Samples of the plasmids are available from the authors.
