# Supplementary material for: NMR Detection and Structural Modeling of the Ethylene Receptor LeETR2 from Tomato
Source: Membranes (Basel). 2022 Jan 18;12(2):107. doi: 10.3390/membranes12020107 (PMC8879215; doi:10.3390/membranes12020107)
Supplement: Supplementary file 1 [file membranes-12-00107-s001.zip › membranes-1516025-supplementary.pdf]

membranes

Article

NMR detection and Structural modelling of the Ethylene Receptor LeETR2 from Tomato

Shukun Wei [1], Yaqing Yang [1], Yuan Yuan [1], Lingyu Du [1], Hongjuan Xue [2] and Bo OuYang [1,*]

1 State Key Laboratory of Molecular Biology, CAS Center for Excellence in Molecular Cell Science, Shanghai Institute of Biochemistry and Cell Biology, Chinese Academy of Sciences; University of Chinese Academy of Sciences, 333 Haike Road, Shanghai 201203, P. R. China; ouyang@sibcb.ac.cn
2 National Facility for Protein Science in Shanghai, ZhangJiang lab, Shanghai Advanced Research Institute, Chinese Academy of Sciences, Shanghai 201210, China;

* Correspondence: ouyang@sibcb.ac.cn (B.O.);

Table S1. Expression conditions of different constructs

Constructs[1]	System[2]	Condition[3]	Expressed[4]	Purified[5]
His8_TrpLE_AtETR1$_{5-116}$	BL21(DE3),	18°C, 0.5mM IPTG	No	No
His8_3C_AtETR1$_{1-116}$	BL21(DE3),	18°C, 0.5mM IPTG	No	No
AtETR1$_{1-116}$_3C_His8	BL21(DE3),	18°C, 0.5mM IPTG	No	No
His8_MBP_3C_AtETR1$_{1-116}$	BL21(DE3),	18°C, 0.5mM IPTG	Yes	No
His8_MBP_3C_AtETR1$_{1-116}$_ΔCys	BL21(DE3),	18°C, 0.5mM IPTG	Yes	No
His8_AtETR2$_{1-148}$_ΔCys	BL21(DE3)	20°C, 0.5mM IPTG	Yes	No
His8_AtERS2$_{1-151}$_ΔCys	BL21(DE3)	20°C, 0.5mM IPTG	No	No
His8_LeETR2$_{1-131}$_ΔCys	BL21(DE3)	20°C, 0.5mM IPTG	No	No
His8_KSI_AtETR2$_{1-148}$_ΔCys	BL21(DE3)	20°C, 0.5mM IPTG	No	No
His8_KSI_AtERS2$_{1-151}$_ΔCys	BL21(DE3)	20°C, 0.5mM IPTG	No	No
His8_KSI_LeETR2$_{1-131}$_ΔCys	BL21(DE3)	20°C, 0.5mM IPTG	Yes	Yes

[1] Designed constructs for expression test.
[2] Expression system for all constructs.
[3] Expression condition used for trials.
[4] Expression results of all constructs.
[5] Purification results of all constructs.

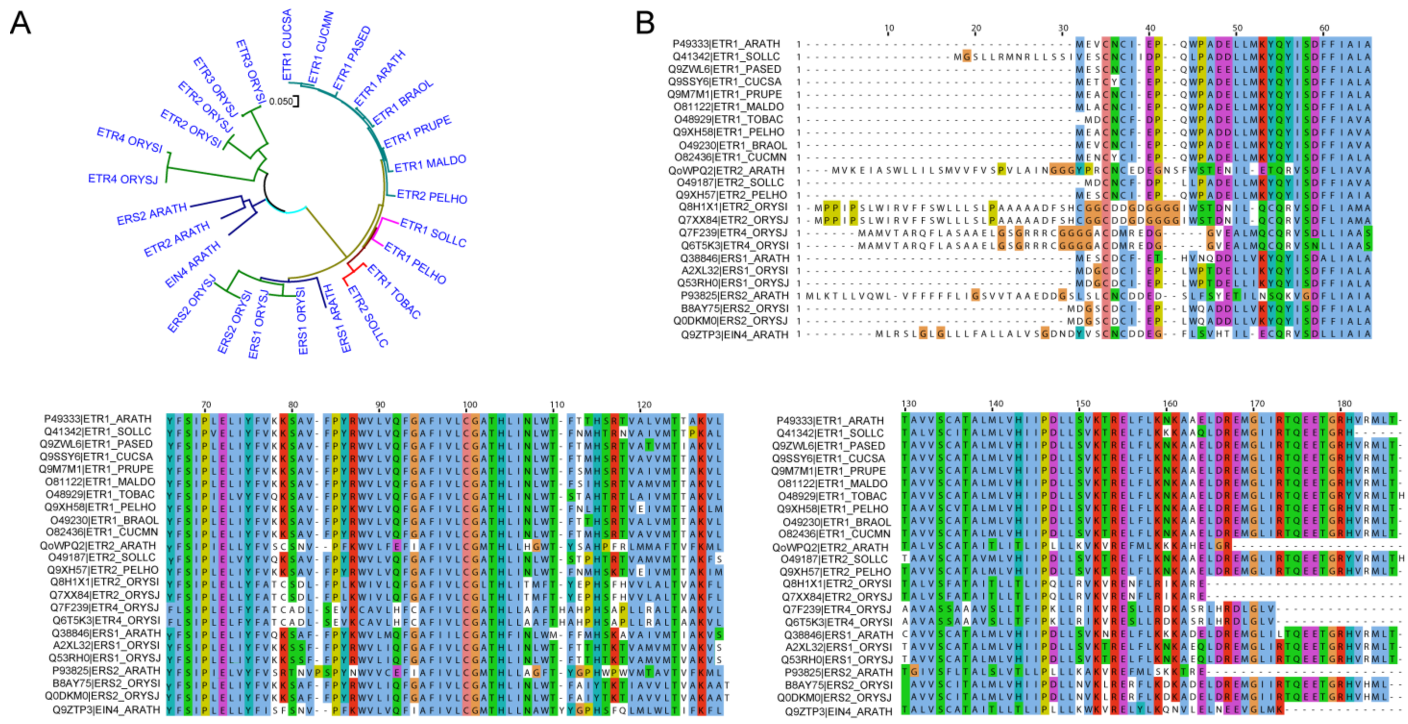

Figure S1. The alignment of ethylene receptor family. (A) The 26 ethylene receptor constructs are used for building up the phylogenetic tree which shows the genetic distance between each other. (B) The sequence alignment of ethylene receptor family for clarity, in which 2 constructs of ETR3-ORYSI and ETR3-ORYSJ are not presented.

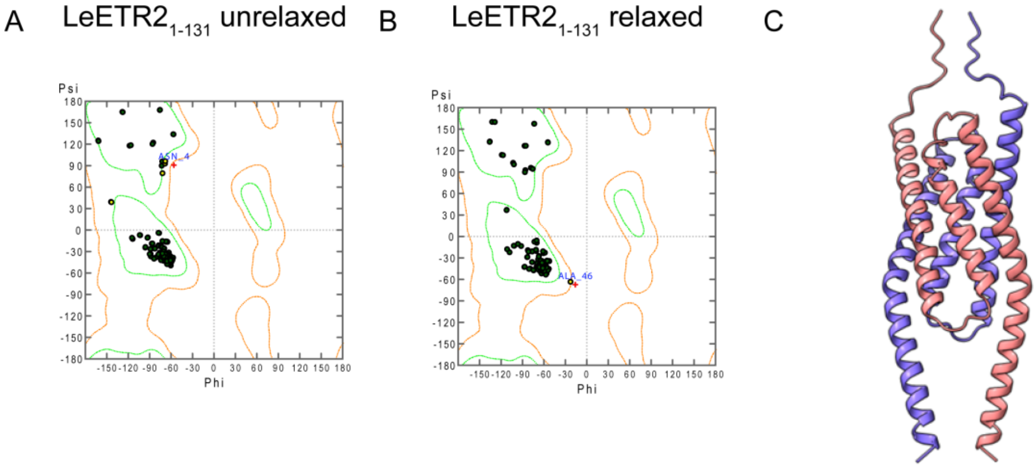

Figure S2. The evaluation of unrelaxed and relaxed models of LeETR2$_{1-131}$ using AlphaFold2 [1]. (A) The ramachandran plot of LeETR2$_{1-131}$ unrelaxed model that shows the outlier residue Asn4 (red crisscrosses with blue annotation) is located in the outlier region while the most residues landed in the most favored core region (green dots), only a few signals landed on the allowed region (yellow dots). (B) The ramachandran plot of LeETR2$_{1-131}$ relaxed model that shows all residues landed in the most favored core region (green dots) except for Ala46 located in the allowed region (Yellow dots). (C) The ranked first relaxed model of LeETR2$_{1-131}$ from AlphaFold2 [1].

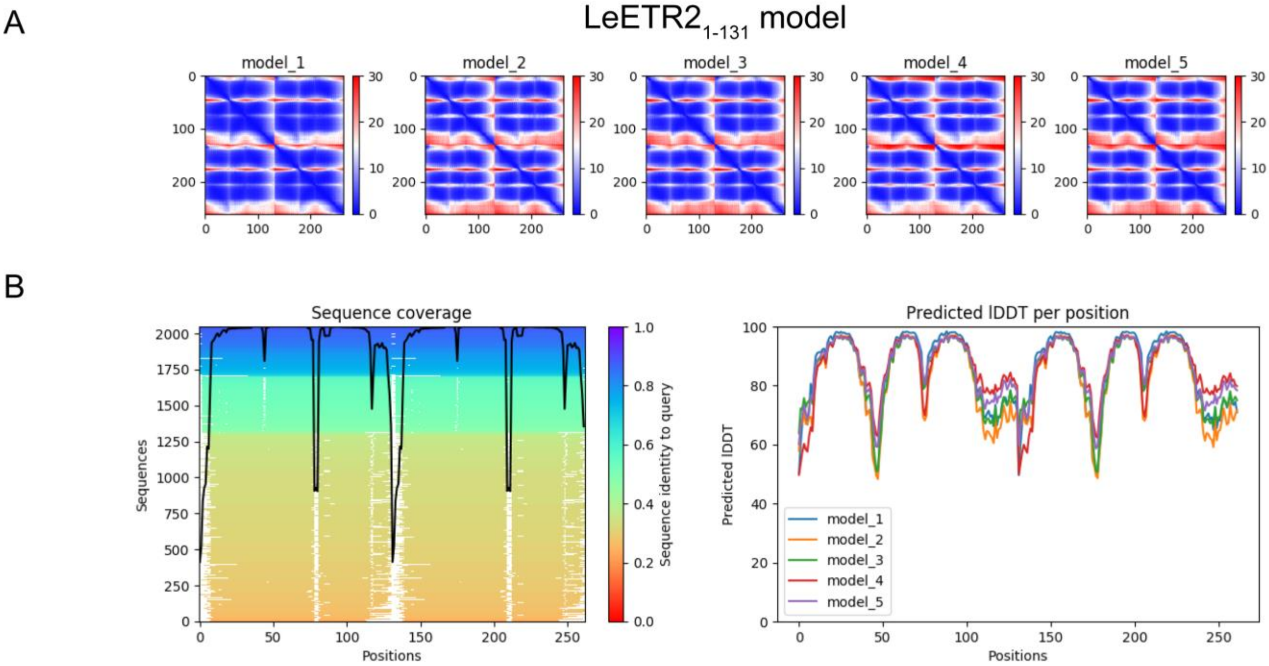

Figure S3. The evaluation of LeETR2$_{1-131}$ models from AlphaFold2**.** (A) To judge the prediction quality we visualize PAE (Predicted Alignment Error) and show the AlphaFold2 confidence measurements. (B) The sequence coverage and sequence identity of AlphaFold2 models of LeETR2$_{1-131}$. The confidence level of core region of model is obviously high. LeETR2 is a three transmembrane-spanning receptor and all these three helices are well posed which are required for latter study. The AlphaFold2 models of LeETR2$_{1-131}$ are qualified using predicted IDDT values. The model 3 is ranked first after relaxation which is used later.

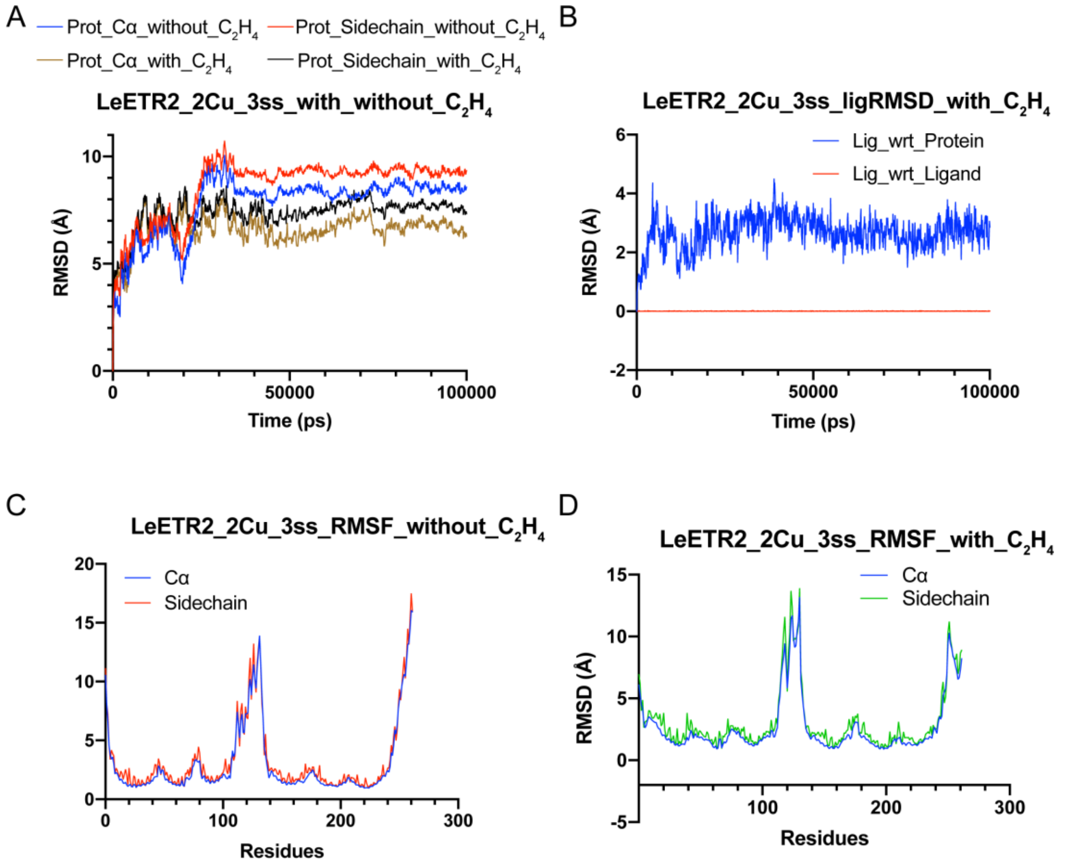

Figure S4. The C*α* and side-chain RMSD of LeETR2$_{1\text{-}131}$, in the absence or presence of ethylene. (A) The C*α* and side-chain RMSD of LeETR2$_{1\text{-}131}$. The protein RMSD with ethylene is lower than that without ethylene which means the entire model is well inhibited in a much more stable conformation. (B) Ligand-RMSD of ethylene for LeETR2$_{1\text{-}131}$. The ethylene itself did not change much and position stayed stably compared with LeETR2$_{1\text{-}131}$ which means the interaction of LeETR2-ethylene is relatively stable at the given sites. (C) The RMSF of LeETR2$_{1\text{-}131}$ without ethylene binding, the C*α* and side-chains fluctuate in a wide range at the N- and C- termini. (D) The RMSF of LeETR2$_{1\text{-}131}$ with ethylene binding, the C*α* and side-chains fluctuate in a narrower range than that of no ethylene binding at the N- and C- termini.

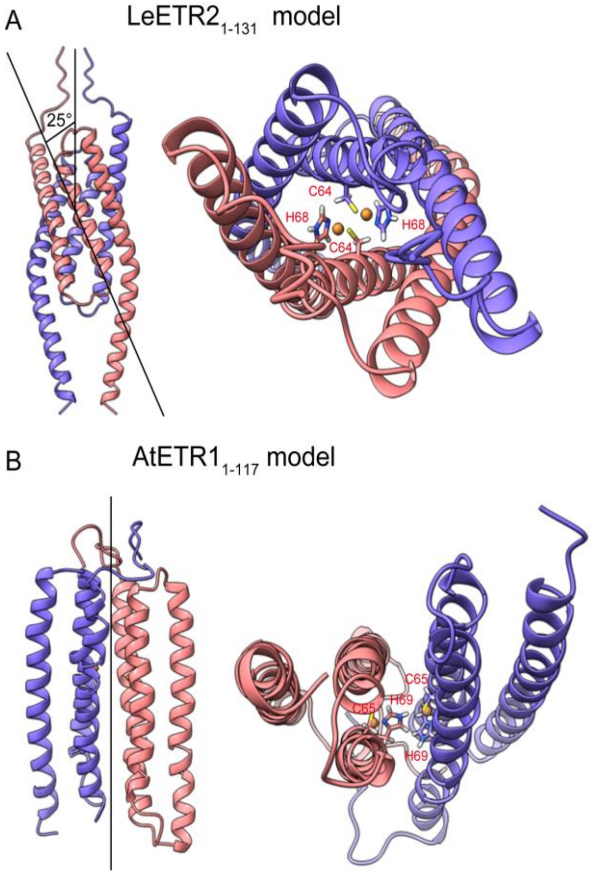

Figure S5. The comparison of LeETR2$_{1\text{-}131}$ and previous model of AtETR1$_{1\text{-}117}$ [2]. (A) The model of LeETR2$_{1\text{-}131}$ is tilted 25 degrees compared with the vertical line. (B) The model of AtETR1$_{1\text{-}117}$ is not tilted compared with the vertical line. The copper binding sites are depicted as sticks and annotated in red.

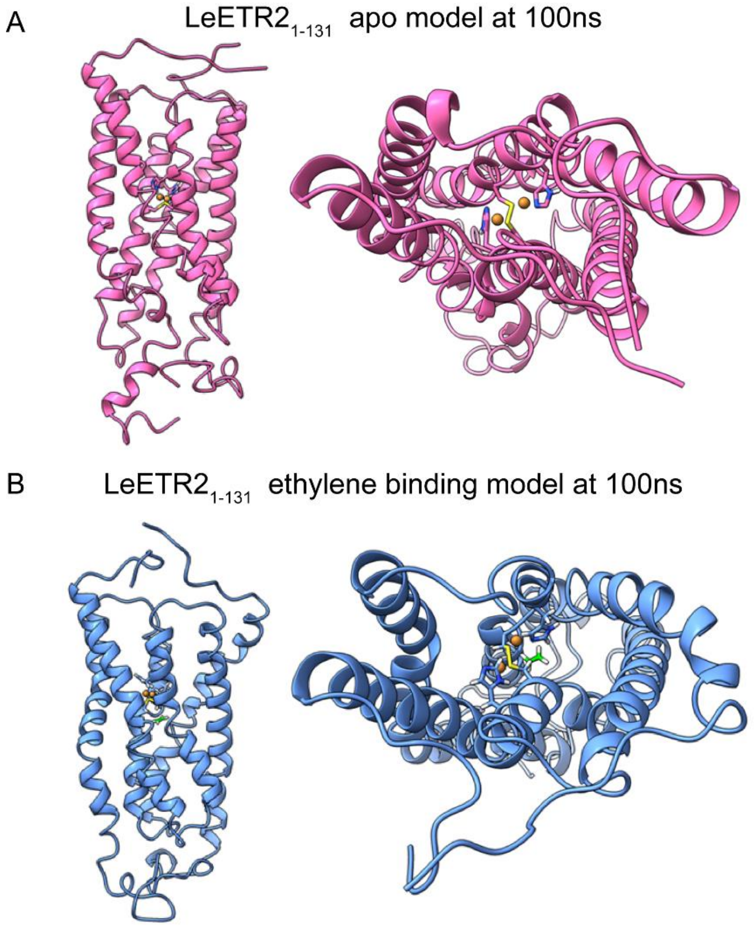

Figure S6. The comparison of LeETR2$_{1-131}$ in the absence or presence of ethylene binding. (A) The apo-state model of LeETR2$_{1-131}$ (Hot pink) at 100 ns of MD simulation run. (B) The ethylene binding model of LeETR2$_{1-131}$ (Cornflower blue) at 100 ns of MD simulation run. The copper ions are depicted as spheres in bronze. The ethylene is depicted as sticks in lime.

References

1. Jumper, J.; Evans, R.; Pritzel, A.; Green, T.; Figurnov, M.; Ronneberger, O.; Tunyasuvunakool, K.; Bates, R.; Zidek, A.; Potapenko, A., et al. Highly accurate protein structure prediction with AlphaFold. *Nature* **2021**, *596*, 583-589, doi:10.1038/s41586-021-03819-2.
2. Schott-Verdugo, S.; Müller, L.; Classen, E.; Gohlke, H.; Groth, G. Structural Model of the ETR1 Ethylene Receptor Transmembrane Sensor Domain. *Scientific reports* **2019**, *9*, 8869, doi:10.1038/s41598-019-45189-w.
